# Editorial: Advancing treatments for protozoan diseases: from resistance mechanisms to novel therapies

**DOI:** 10.3389/fmicb.2026.1857081

**Published:** 2026-05-05

**Authors:** María C. Touz, David Arranz-Solís, Sheila Nardelli

**Affiliations:** 1 Instituto de Investigación Médica Mercedes y Martín Ferreyra, Consejo Nacional de Investigaciones Científicas y Técnicas (INIMEC-CONICET), Universidad Nacional de Córdoba, Córdoba, Argentina; 2 SALUVET, Department of Animal Health, Faculty of Veterinary Sciences, Complutense University of Madrid, Madrid, Spain; 3 Instituto Carlos Chagas, Fundação Oswaldo Cruz, Curitiba, Brazil

**Keywords:** antiparasitic resistance, drug repurpose, molecular targets, One Health, protozoan diseases

Protozoan diseases remain a major yet often underappreciated global health burden, disproportionately affecting vulnerable populations and contributing to significant morbidity, mortality, and socioeconomic impact. Despite decades of research, effective control of diseases such as malaria, Chagas disease, leishmaniasis, and giardiasis continues to be challenged by the limited availability of vaccines, the toxicity and inefficacy of current treatments, and the alarming rise of antiparasitic drug resistance.

This Research Topic brings together a diverse collection of contributions addressing these challenges, spanning mechanistic studies, drug discovery, and translational perspectives. In line with the broader context of antimicrobial resistance as a global threat, these works highlight the urgent need for innovative therapeutic strategies against protozoan parasites.

A central theme of this Research Topic is the identification of novel molecular targets and regulatory mechanisms. In an Original Research article, Alonso et al. investigate bromodomain factor 3 (TcBDF3) in *Trypanosoma cruzi*, identifying 1,3,4-oxadiazoles as promising inhibitors with trypanocidal activity. Complementing this, Níttolo et al. characterize the RNA-binding protein TcSR62 as a potential therapeutic target and demonstrate the repurposing potential of sorafenib tosylate. In *Plasmodium falciparum*, Wunderlich et al. provide transcriptomic insights into iron transport pathways, revealing key proteins involved in parasite iron homeostasis and identifying potential targets for antimalarial strategies. In the same organism, Chakraborty et al. evaluate the role of the phosphatidylethanolamine-binding protein PfPEBP using CRISPR/Cas9-mediated gene disruption. Their comprehensive phenotypic analysis demonstrates that PfPEBP is expressed throughout the intraerythrocytic life cycle but is dispensable for asexual growth, gametocyte development, and gametogenesis under *in vitro* conditions.

Several contributions focus on epigenetic regulation and cellular processes in parasite survival and drug response. In an Original Research article, Patolsky et al. demonstrate the essential role of protein acetylation in *Giardia lamblia* and highlights garcinol as a potent giardicidal compound capable of inducing apoptosis-like processes and preventing parasite recrudescence. Similarly, Barsola et al. explore the cytotoxic effects of ivermectin in *G. lamblia*, showing induction of oxidative stress, DNA damage, and cell cycle arrest.

Another major focus of this Research Topic is the development and repurpose of therapeutic compounds. Maciel et al. report that the antifungal drug nystatin exhibits significant trypanocidal activity against *T. cruzi*, including synergistic effects with benznidazole. Likewise, García-Bustos et al. demonstrate the antiparasitic potential of Amazonian plant extracts against *G. lamblia*, identifying apoptosis induction and oxidative stress as key mechanisms and showing synergy with metronidazole.

Innovative therapeutic strategies based on targeted peptides and selective parasite killing are also represented. Iniguez et al. evaluate ligand-lytic peptide constructs targeting *Leishmania major* and *T. cruzi*, demonstrating selective antiparasitic activity with minimal host toxicity.

The Research Topic further includes studies addressing drug resistance mechanisms and genetic adaptation. Gu et al. investigate the genomic basis of monensin resistance in *Eimeria tenella*, identifying candidate mutations associated with resistance development. In a complementary Perspective article, Sharma et al. discuss the role of autophagy in *P. falciparum* as a mechanism contributing to antimalarial drug resistance, highlighting the survival strategies of parasites under drug pressure.

In addition to experimental studies, this Research Topic includes reviews and epidemiological analyses that provide broader context. Rivero et al. present a comprehensive overview of *Cryptosporidium spp*. in Argentina, emphasizing epidemiology, transmission, and research gaps from a One Health perspective. Similarly, Carrer et al. analyze the epidemiology and treatment challenges of cutaneous leishmaniasis, focusing on the potential of topical therapies as less toxic and more accessible alternatives.

A Brief Research Report by Larrazabal et al. further contributes to this Research Topic by demonstrating the role of host cholesterol transport via NPC1 in the replication of *Besnoitia besnoiti*, reinforcing the importance of host–parasite metabolic interactions as therapeutic targets.

Collectively, the studies in this Research Topic underscore the complexity of protozoan diseases and highlight multiple complementary strategies to address them. These include targeting essential molecular pathways, repurposing existing drugs, developing novel compounds, and leveraging interdisciplinary approaches such as genomics, bioinformatics, and One Health frameworks.

We thank all authors and reviewers for their valuable contributions and hope that this Research Topic will stimulate further research and foster collaborations aimed at advancing the understanding and treatment of protozoan diseases.

